# Supplementary material for: Feature selection based on differentially correlated gene pairs reveals the mechanism of IFN-β therapy for multiple sclerosis
Source: PeerJ. 2020 Mar 16;8:e8812. doi: 10.7717/peerj.8812 (PMC7081782; doi:10.7717/peerj.8812)
Supplement: Supplemental Information 2 [file peerj-08-8812-s002.docx]

**Supplementary file 2: Further validation using another microarray dataset**

**Experimental data**

To further justify our proposed procedure, we applied it to another microarray experiment dataset (i.e., GSE41846 in GEO database). There were 318 arrays in this experiment, which enrolled 62 untreated MS patients and 58 MS patients being treated with IFN. The expression values were measured three times at three consecutive years. Here, no pre-processing had been performed, the expression matrix deposited in GEO database was used directly.

**Results**

With the cutoff for the absolute difference of SCCs between the treated group and non-treated group being set at 0.6, the proposed method identified 2842 consistently differentially correlated edges (DCEs). Among them, 2395 had an adjusted p-value/a FDR <0.01. Given the sample size of this training set is large (with three time points), we further refined the identified DCEs by choosing a more stringent cutoff, say, 0.8. At this cutoff, there were 206 DCEs, and 203 statistically significant DCEs involving 101 unique genes were included (with a FDR<0.01). Based on these, we anticipate that either the corresponding network should be highly connected (thus dense) or at least several genes have extremely high connectivity values such as CCL2 which has a highest connectivity value of 78. According to the GeneCards database, other than 6 non-recognized genes, 42 genes were directly related to MS and 53 genes were indirectly related to MS. CCL2, SP140, STAT1, NOD1 and SOCS1 were ranked on the top of this directly-related-to-MS list. Meanwhile, the Interferome database (<http://www.interferome.org/>) indicated 96 of them were associated with IFN.

In summary, with this validation we have demonstrated that the proposed procedure can indeed identify genes that are biologically relevant to MS or IFN, and that the sample size does matter. Hence, with a large longitudinal dataset a stringent cutoff value for the absolute difference of SCCs may be employed while with a small longitudinal dataset a less stringent cutoff should be considered.
